# Neuroregulatory and clinical efficacy of auricular vagus nerve stimulation in elderly patients with chronic insomnia comorbid with functional dyspepsia: protocol for a randomized controlled trial

**DOI:** 10.3389/fmed.2025.1537515

**Published:** 2025-03-18

**Authors:** Hao Zhou, Xing Tang, Dan Wang, Zubo Huang, Yue Zeng, Shanshan Liu, Chao Wang

**Affiliations:** ^1^Sichuan Clinical Research Center for Sub-Health, Sichuan Integrative Medicine Hospital, Chengdu, China; ^2^School of Acupuncture-Moxibustion and Tuina, Chengdu University of Traditional Chinese Medicine, Chengdu, China; ^3^College of Acupuncture and Tuina, Chongqing University of Chinese Medicine, Chongqing, China

**Keywords:** auricular, vagus nerve stimulation, elderly patients, chronic insomnia, functional dyspepsia, randomized controlled trial

## Abstract

**Objective:**

This study innovatively employs transcutaneous auricular vagus nerve stimulation (taVNS), a non-invasive physical therapy, to intervene in elderly patients with chronic insomnia (CI) comorbid with functional dyspepsia (FD). Through systematic investigation of the molecular mechanisms underlying vagus nerve pathway regulation in ameliorating intestinal inflammatory microenvironment and modulating central neurotransmitter homeostasis, this research aims to provide a novel, neuromodulation-based precision therapeutic approach characterized by favorable safety and tolerability for integrated management of geriatric comorbidities.

**Methods/design:**

This double-blind randomized controlled trial will enroll 60 elderly patients (60–85 years) meeting ICSD-3 criteria for CI and Rome IV criteria for FD. Using block randomization with computer-generated sequences, eligible participants will be allocated 1:1 to either active taVNS group (*n* = 30) or sham control group (*n* = 30). The CFDA-certified transcutaneous vagus nerve stimulator (Model tVNS501, Reach Medical, China; Registration No. SuXieZhun20212090050) will be positioned at standardized anatomical sites: the concha cymba (the inferior margin of the intersection between the superior and inferior crura of the antihelix within the cymba conchae), electrical stimulation will deliver with fixed parameters (frequency: 80 Hz, pulse width: 100 μs, pulse 40–60s). The active group will receive validated taVNS parameters, while the sham group will undergo identical procedures without electrical output. Interventions will be administered 30 min per session, 5 sessions weekly for 3 consecutive weeks. Primary endpoints include changes in Pittsburgh Sleep Quality Index (PSQI) and Nepean Dyspepsia Symptom Index (NDSI) at week 3. Secondary outcomes encompass Insomnia Severity Index (ISI), 36-Item Short Form Survey (SF-36), and serum biomarkers (pro-inflammatory cytokines IL-1*β*, IL-4, IL-6, TNF-*α*, hs-CRP, TGF-β; neurotransmitters Dopamine (DA), serotonin (5-hydroxytryptamine, 5-HT), norepinephrine (NE), Glutamate (Glu), *γ*-aminobutyric acid (GABA)). Safety profiles will be systematically evaluated using CTCAE v5.0 criteria, with all adverse events documented throughout the study.

**Discussion:**

This study mitigate the adverse effects associated with the significant side effects of oral medications in elderly patients with CI comorbid with FD. It seeks to scientifically validate the clinical efficacy of taVNS therapy, elucidate its underlying anti-inflammatory and neuromodulatory mechanisms, and establish a multimodal evidence chain integrating “efficacy-inflammation-neuromodulation.” By doing so, this research provides a novel, convenient, scientifically validated, effective, and safe non-pharmacological therapeutic approach for elderly patients with CI and FD, it offers innovative insights and methodologies for the development of pharmaceuticals, medical devices, and related products.

## 1 Background

According to the 2022 Statistical Bulletin of National Economic and Social Development released by National Bureau of Statistics of China, by the end of 2022, the population aged 60 years and above in China had reached 280 million, accounting for 19.8% of the total population, among which 210 million were aged 65 years and above, representing 14.9% of the population. The “14th Five-Year Plan” for Healthy Aging issued by National Health Commission of the PRC highlights the concerning health status of the elderly population, with over 78% suffering from at least one chronic disease. This necessitates a shift in elderly healthcare services from a single-disease model to a multi-morbidity management model. The Blue Book of Aging Society: Report on Aging Society Development (2022) published by the China Research Center on Aging reveals that over 180 million elderly individuals in China are affected by chronic diseases, with an average duration of life with chronic diseases of 9.1 years among those aged 60 and above. Moreover, two-thirds of individuals aged 65 and above suffer from multiple coexisting conditions.

In terms of disease prevalence, chronic diseases are highly prevalent among the elderly, and more than half experience poor sleep quality. Studies indicate (1–6) that approximately 10–30% of adults worldwide suffer from insomnia, with the prevalence among the elderly (≥65 years) ranging from 30 to 50%. In China, the incidence of insomnia has reached 38.2%, affecting over 300 million individuals, and the prevalence of sleep disorders among the elderly is 46.0%.

The expert consensus on the diagnosis and treatment of functional dyspepsia in the elderly reports that the global prevalence of dyspepsia is approximately 20%, with rates in Asia ranging from 8 to 23%, and the prevalence among Chinese elderly ranging from 18 to 35% (7). CI comorbid with FD is a highly prevalent clinical manifestation. Research shows (8) that up to 79.48% of insomnia patients exhibit gastrointestinal symptoms, while nearly 70% of elderly individuals with dyspepsia experience comorbid insomnia. Dyspepsia and insomnia often coexist, and sleep quality is closely associated with the severity and prognosis of dyspepsia. Patients with dyspepsia and impaired sleep quality tend to exhibit more severe symptoms and poorer prognoses (9, 10).

Currently, pharmacological treatments for CI in the elderly are associated with systemic risks. Studies have shown (11–14) that the risk of dependence on benzodiazepines is 34.7% (OR = 3.2, 95%CI 2.1–4.9), and the incidence of withdrawal syndrome after discontinuation is 28.4% (RR = 2.8). Additionally, a significant dose ineffectiveness threshold effect has been observed, necessitating off-label dosing in some patients, while 12.7% of patients exhibit a decline in estimated glomerular filtration rate (eGFR), and the incidence of hepatorenal toxicity is ≥30%. The treatment of FD lacks targeted therapies and effective drugs, with proton pump inhibitors (PPIs) showing a clinical response rate of only 38.2% (95%CI 32.7–44.1), and long-term use of prokinetic agents increases the risk of QT interval prolongation (HR = 1.68) (15, 16).

When CI and FD coexist, the risk of drug interactions increases by 71% (*p* < 0.001) (17), and the cost-effectiveness ratio of conventional therapies is as high as $152,300 per quality-adjusted life year (QALY) (18). Therefore, there is an urgent need for reliable, safe, cost-effective, and efficient clinical treatment options for elderly patients with CI comorbid with FD. In this context, taVNS emerges as a novel therapeutic approach, offering a potential innovative solution for this patient population.

This study will systematically evaluate the clinical efficacy of transcutaneous auricular vagus nerve stimulation (taVNS) in elderly patients with CI comorbid with FD. By analyzing dynamic fluctuations in inflammatory cytokines and neurotransmitters, we will objectively validate the clinical effectiveness of taVNS in geriatric comorbidity management for the first time. The investigation will further elucidate the vagus nerve-mediated regulatory mechanism of duodenal mucosal inflammation and establish a novel non-pharmacological intervention characterized by precision targeting, high safety profile, and enhanced therapeutic compliance, which will provide a theoretical foundation for developing neuromodulation-based integrated therapeutic strategies for elderly patients with multimorbidity management.

## 2 Methods/design

### 2.1 Settings and subjects

This randomized controlled trial (RCT) was conducted at the Sichuan integrative medicine hospital (Chengdu, Sichuan, China). The study protocol (Version 2.0, dated March 14, 2024) strictly adhered to the ethical principles of the Declaration of Helsinki and was approved by the Clinical Research Ethics Committee of the Sichuan Provincial Hospital of Integrated Traditional Chinese and Western Medicine (Approval No.: KY-2024-001; Date: March 18, 2024).

In accordance with the International Standards for Clinical Trial Registration (ICTRP), the research team prospectively registered the trial at the Chinese Clinical Trial Registry on February 2, 2024 (Website: www.chictr.org.cn; Registration No.: ChiCTR2400080630).

In compliance with ethical requirements, researchers provided detailed explanations of the study objectives, procedures, and potential risks to all participants. Informed consent was obtained from each participant after ensuring their full understanding of the study, and participants retained the right to withdraw unconditionally at any time. The first participant was formally enrolled on July 26, 2024, marking the commencement of the study’s implementation phase.

### 2.2 Diagnostic criteria

Participants must meet both international diagnostic standards: Chronic Insomnia (ICSD-3) (19) and Functional Dyspepsia (Rome IV) (20).

### 2.3 Inclusion criteria

*Age and gender*: Participants aged between 60 and 85 years, regardless of gender.

*Clinical presentation*: Patients exhibiting a chronic, recurrent condition that meets the diagnostic criteria, with no evidence of structural diseases that could explain the aforementioned symptoms.

*Diagnostic tests*: Completion of electronic gastroscopy or upper gastrointestinal radiography, blood biochemistry, and stool routine tests within the past year, with results confirming the absence of structural abnormalities, ensuring the exclusion of other diseases causing dyspeptic symptoms, and ruling out organic digestive system diseases, metabolic or systemic diseases. There should be no evidence of chronic gastritis with erosion, ulcers, tumors, or other organic lesions.

*Medication and MRI contraindications*: No intake of gastrointestinal motility drugs, antacids, or sleeping pills within the past week. Absence of metal implants or claustrophobia, which are contraindications for MRI.

*Informed consent*: Participation must be based on genuine personal willingness, adherence to the clinical trial design, and the signing of an informed consent form.

### 2.4 Exclusion criteria

*Systemic diseases*: Patients with severe psychiatric disorders, severe cardiovascular or cerebrovascular diseases, hepatic or renal dysfunction, digestive dysfunction, coagulation disorders, or other severe systemic diseases.

*Medical history*: Individuals with a history of gastrointestinal surgery, psychiatric disorders, alcohol abuse, or drug abuse. Patients with overlapping syndromes such as Gastroesophageal Reflux Disease (GERD) or Irritable Bowel Syndrome (IBS), or those with current *Helicobacter pylori* infection.

*Systemic or environmental factors*: Patients experiencing insomnia or dyspepsia caused by systemic conditions such as pain, fever, cough, surgery, trauma, or external environmental factors.

*Ear skin conditions*: Patients with skin conditions in the ear stimulation area that may affect conductivity, including allergies, abrasions, ulcers, edema, neoplasms, scars, inflammation, or papules.

### 2.5 Sample size estimation

Based on existing RCT literature, the efficacy rate of taVNS in treating elderly patients with CI ranges from 73.3 to 93.3% (21, 22), while its efficacy in FD reaches 92.3% (3). Building upon our preliminary exploratory clinical study involving 10 cases, we observed an 80.0% efficacy rate (*p* < 0.01) for taVNS in treating elderly patients with CI comorbid with FD, demonstrating significant clinical effectiveness.

This study employed a superiority trial design and utilized PASS 2021 software (NCSS LLC, Kaysville, USA) for prospective sample size estimation. Based on clinical data reported in reference (23), with anticipated efficacy rates of 20% in the sham stimulation group and 70% in the active stimulation group, we conducted a two-independent proportion *z*-test analysis with the following parameters: type I error probability *α* = 0.05, statistical power 1-*β* = 0.99, and 1:1 allocation ratio. Through Monte Carlo simulation validation, the minimum theoretical sample size was calculated as 28 participants per group.

In accordance with CONSORT Statement guidelines regarding dropout rates and considering our institutional clinical practice experience with anticipated 5–10% dropout rates, we ultimately determined to enroll 30 participants per group (total *N* = 60). This sample size enables detection of a Cohen’s *h* effect size of 0.99 (Power = 0.991, 95% CI 0.89–1.07), satisfying the statistical power requirements for superiority trials.

### 2.6 Randomization and blinding

The randomized controlled trial (RCT) utilized a simple randomization method implemented via the PROC PLAN procedure in SAS 9.4 software, which was designed by an independent statistician. A fixed seed number of 60 was preset to ensure reproducibility, and participants were allocated to the two groups (30 each) at a 1:1 ratio, with random numbering from 01 to 60, generating 60 unique sequences to ensure unbiased group allocation. The group allocation information was sealed in tamper-evident opaque envelopes and managed by a blinded study coordinator who was not involved in participant recruitment or outcome assessment, ensuring strict allocation concealment.

During trial execution, consecutively enrolled participants meeting inclusion criteria were sequentially assigned unique identification numbers. The blinded trial coordinator strictly adhered to the enrollment chronology when unsealing pre-sequenced, tamper-evident sealed envelopes. The revealed allocation information was subsequently transferred to an independent investigator uninvolved in recruitment or intervention delivery, who executed group assignments per the predefined randomization matrix. The study protocol explicitly prohibited any deviation from allocation sequences or non-programmatic modifications.

**Figure 1:**
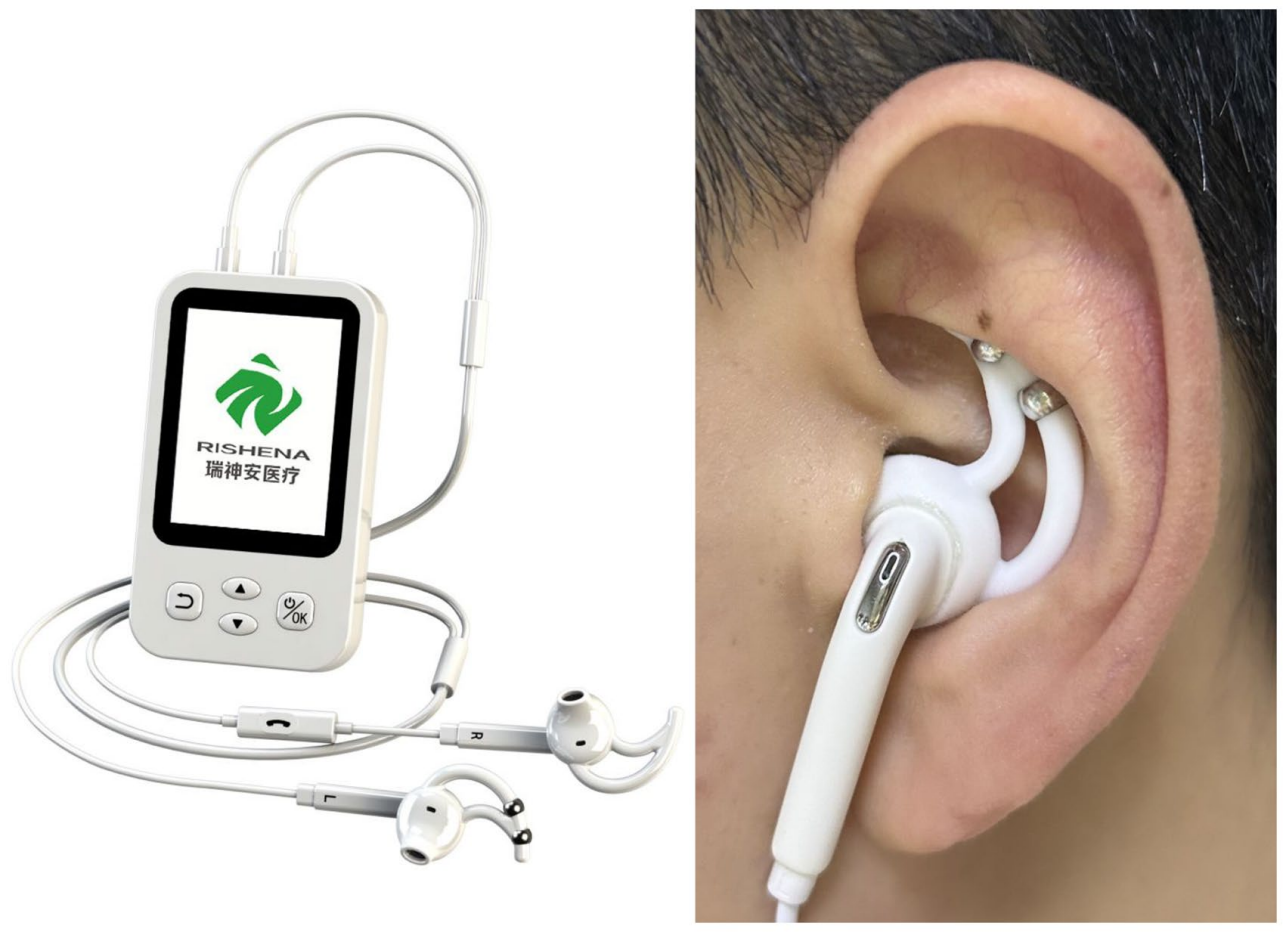
TaVNS Stimulation Device and Example Diagram of Electrode Placement.

The independent researcher, based on randomized allocation, preset the transcutaneous auricular vagus nerve stimulator (tVNS501) with fixed parameters (intensity: 20–40 mA, frequency: 80 Hz, pulse width: 100 μs, pulse 40–60s) and blinded the display screen using opaque tape before handing it to a clinical operator. In the active stimulation group, the device was powered to deliver real stimulation, while in the sham stimulation group, the device remained inactive (no current output) but maintained physical contact. A triple-blind design (participant-operator-outcome assessor) was implemented throughout the study to maintain blinding of group assignment (taVNS or sham taVNS). Unblinding was performed post-database lock by an independent statistician using SAS 9.4 (SAS Institute Inc., Cary, NC), strictly adhering to the predefined protocol. This study strictly adhered to the principle of observer independence in clinical research (Figure 1).

### 2.7 Intervention

Following standardized screening, all participating clinicians underwent unified training in both taVNS and sham taVNS protocols. The informed consent form explicitly stated that the study involved two distinct neuromodulation interventions (which may differ in electrical sensation and therapeutic responses). Training modules included: (1) Device operation: standardized procedures for power cycling and locked parameter interfaces; (2) Anatomical localization: precise electrode placement at the tragus: the inferior margin of the intersection between the superior and inferior crura of the antihelix within the cymba conchae and the junction of the inferior crus of the antihelix and the auricle within the cymba conchae using auricular anatomical charts; (3) Blinding maintenance: standardized communication protocols to prevent unintentional disclosure of group allocation. All interventions were conducted in standardized treatment rooms within the hospital inpatient department.

All clinicians participating in this study were required to have at least 2 years of standardized acupuncture practice. Participants were placed in a supine position, and the auricular stimulation areas were cleaned with 75% ethanol solution followed by placement of ear clips. The clinically trained operator, blinded to group assignment, using transcutaneous auricular vagus nerve stimulators (tVNS501), precisely placed bipolar electrodes at standardized anatomical sites: the concha cymba (the inferior margin of the intersection between the superior and inferior crura of the antihelix within the cymba conchae). The intervention protocol consisted of 30-min sessions, administered weekdays from 9:00 to 11:00 AM for three consecutive weeks (15 sessions total).

Participants shall be withdrawn from the trial and provided with appropriate medical interventions under any of the following circumstances: (1) occurrence of serious adverse events; (2) voluntary withdrawal request from the participant; (3) clinical deterioration of the underlying condition; (4) requirement for prioritized treatment of other medical disorders; or (5) development of localized irritation or allergic reactions.

### 2.8 Clinical evaluation system

The clinical evaluation utilized PSQI, ISI, NDSI, and SF-36. As evidenced by prior studies (24–27), all instruments underwent systematic cultural adaptation and linguistic validation, and have been successfully utilized in Chinese clinical practice for years: PSQI has been widely adopted in insomnia assessment, ISI demonstrated robust validity in gastrointestinal disease research, NDSI exhibited specific discriminative capacity for dyspeptic symptoms in Chinese populations, and the Chinese version of SF-36 showed consistent reliability and validity in evaluating quality of life among elderly populations, collectively ensuring measurement reliability.

#### 2.8.1 Baseline characteristics

Data were collected using a standardized Case Report Form (CRF), including:

Demographic characteristics: Sex, age, educational attainment, and occupational category.

Clinical characteristics: Disease duration (months), marital status, and dietary patterns.

Anthropometric measurements: Body mass index (BMI, kg/m^2^).

#### 2.8.2 Primary outcome measures

##### 2.8.2.1 Pittsburgh sleep quality index (PSQI)

Assessment timepoints: Baseline (T0), post-intervention (T1, week 3), and follow-up (T2, week 4 post-treatment).

Evaluation domains: Seven components including subjective sleep quality, sleep latency, and sleep duration.

Scoring system: Total scores range from 0 to 21, with ≥8 indicating sleep disturbance. A change ≥3 points was considered clinically significant.

##### 2.8.2.2 Nepean dyspepsia symptom index (NDSI)

Assessment timepoints: Consistent with the aforementioned schedule (T0-T2).

Symptom assessment: Four core symptoms (early satiety, postprandial fullness, etc.) were quantified according to Rome IV criteria.

Scoring system: Each item was scored 0–4 (total range 0–16). A symptom reduction rate ≥ 30% indicated clinical effectiveness.

#### 2.8.3 Secondary outcome measures

##### 2.8.3.1 Insomnia severity index (ISI)

Assessment timepoints: Consistent with the aforementioned schedule (T0-T2).

Multidimensional assessment: Five clinical dimensions were evaluated, including sleep disturbance severity and daytime functional impairment.

Clinical thresholds: 0–7 (no clinically significant insomnia), 8–14 (subclinical insomnia), 15–21 (moderate-to-severe insomnia).

##### 2.8.3.2 36-item short form health survey (SF-36)

Assessment timepoints: Consistent with the aforementioned schedule (T0-T2).

Health domains: Eight dimensions including physical functioning (PF) and role limitations due to physical health (RP).

Score transformation: Raw scores were converted to standardized scores (0–100) using the formula: [(Raw score - Minimum possible score)/(Maximum possible score - Minimum possible score)] × 100.

##### 2.8.3.3 Inflammatory biomarkers

Assessment timepoints: Consistent with the aforementioned schedule (T0-T1).

Analytical method: Serum concentrations of IL-1*β*, IL-4, IL-6, TNF-*α*, hs-CRP, and TGF-β were quantified using enzyme-linked immunosorbent assay (ELISA).

Blood sampling protocol: Venous blood samples were collected at T0 (baseline) and T1 (post-intervention) under morning fasting conditions (≥8 h overnight fast).

Interpretation criteria: Concentrations were reported in pg/mL. Biological significance was defined as >15% change from baseline.

##### 2.8.3.4 Neurotransmitter metabolomics profiling

Assessment timepoints: Consistent with the aforementioned schedule (T0-T1).

Analytical platform: Separation was performed using an Agilent 1,290 Infinity LC system coupled with a 5,500 QTRAP mass spectrometer (SCIEX) operated in negative ion mode.

Blood sampling protocol: Venous blood samples were collected at T0 (baseline) and T1 (post-intervention) under morning fasting conditions (≥8 h overnight fast).

Analytes:

1. Monoamines: DA, 5-HT, NE.
2. Amino acids: Glu, GABA.

Interpretation criteria: Pearson correlation analysis was performed to assess the association between dynamic changes in neurotransmitter levels and improvement magnitudes of PSQI/NDSI scores, with |*r*| ≥ 0.3 defined as significant correlation (two-tailed test, *p* < 0.05).

#### 2.8.4 Blood sample processing

Venous blood was collected using 5 mL PET separator gel tubes with clot activator (Jiangsu Kangjian Medical Products Co., Cat.No. 0105–0512). Certified phlebotomists performed venipuncture between 08:00–09:00 under fasting conditions (≥8 h fasting), with blood volume strictly controlled at 5.0 ± 0.5 mL. Tubes were immediately labeled with subject-specific identifiers: unique study ID (STID), group allocation (A/B), and collection timestamp (YYYY-MM-DD HH:MM) on both tube body and cap. Samples were transported to the clinical laboratory within 10 min, allowed to clot at room temperature (22 ± 1°C) for 30 min, then centrifuged at 3,000 × g for 15 min at 4°C using a Shuke Benchtop Low-Speed Refrigerated Centrifuge (Sichuan Shuke Instrument Co., Model TDL-6). The supernatant was aliquoted into 2 mL cryovials (1.0 mL for inflammatory biomarkers and 2.0 mL for neurotransmitter profiling). All aliquots were flash-frozen in liquid nitrogen and stored at −80°C (Qingdao Haier Biomedical Co., Model DW-86 L828).

#### 2.8.5 Statistical analysis

Primary analyses were conducted using the intention-to-treat (ITT) dataset, defined as the subset of participants who completed ≥1 intervention session, baseline assessment, and ≥ 1 post-baseline evaluation. In accordance with CONSORT guidelines, missing data were handled via multiple imputation (R package ‘mice’ v3.14.0). Sensitivity analyses were performed using the per-protocol set (PPS) to validate robustness.

The per-protocol set (PPS) followed ICH E9 guidelines with three integrated criteria: protocol compliance requiring 80–120% intervention dose completion, avoidance of prohibited medications, and key visit time deviations ≤3 days; data integrity enforcing <5% missingness in CDISC ODM v2.1-compliant CRF fields and laboratory CV% <15; and analytical scope restrictions that included cases with complete demographic/clinical data for baseline analysis, excluded efficacy cases with blinded-independent-adjudication-confirmed major protocol deviations (MPDs), while employing a modified PPS (mPPS) for safety analysis to retain participants discontinuing due to adverse events (AEs).

Post-trial, an independent third-party observer (uninvolved in study design or intervention delivery) performed the following: (1) Baseline data collection: Including gender, age, disease duration (months), BMI, education level, occupation category, marital status, and dietary patterns; (2) Outcome measures collection: Primary outcomes: Pittsburgh Sleep Quality Index (PSQI) and Nepean Dyspepsia Symptom Index (NDSI);Secondary outcomes: Insomnia Severity Index (ISI), 36-Item Short Form Health Survey (SF-36), inflammatory biomarkers (e.g., IL-6, CRP), and neurotransmitter levels (e.g., 5-HT, GABA); (3) Data de-identification: Removal of all direct or indirect identifiers (e.g., name, ID number), with assignment of unique study IDs.

After de-identified data underwent ETL (Extract-Transform-Load) processing for standardization, the standardized data were imported into statistical software for further analysis. Measurement data were expressed as Mean ± SD. Differences among groups were analyzed by Student *t* test or one-way ANOVA, SNK or LSD method was used for multiple comparison. Qualitative data were described as percentages and analyzed using Chi-square (*χ*^2^) test or Fisher’s exact test as indicated. The *p*-value reported was two-sided and value of less than 0.05 was considered statistically significant. All analyses were performed using the SPSS software (Version 20.0, SPSS Inc., USA).

### 2.9 Safety monitoring and adverse event reporting

#### 2.9.1 Observation periods

Three-tiered safety observation windows:

Single-session phase: From intervention initiation to 24 h post-procedure.

Full intervention phase: From first treatment to T1 follow-up.

Extended follow-up phase: T1 to T2 follow-up.

#### 2.9.2 AE classification and reporting

AE documentation: All adverse events graded (Grade 1–5) per CTCAE v5.0 criteria.

SAE reporting: Defined as events causing death, life-threatening conditions, hospitalization, or permanent disability, requiring immediate (<24 h) reporting to Ethics Committee of Sichuan integrative medicine hospital.

Causality assessment: WHO-UMC causality categories applied (certain/probable/possible/unrelated).

#### 2.9.3 Statistical analysis

Incidence rate: AE incidence = (Number of AE episodes / Total treatment sessions) × 100%.

Severity stratification:

- Grade 1–2: Mild, requiring no intervention.
- Grade ≥ 3: Mandating medical intervention or protocol modification.

Temporal analysis: Time-to-event analysis using Cox proportional hazards model.

### 2.10 Data safety monitoring protocol

An independent Data Safety Monitoring Board (DSMB) was established, comprising clinical specialists, biostatisticians, methodological experts, and ethicists (all members provided signed conflict-of-interest declarations). The DSMB conducts dynamic safety surveillance through scheduled 24-week meetings and emergency sessions triggered by predefined thresholds (e.g., >10% SAE incidence at any site or > 20% participant dropout rate).

Adverse events were classified into four tiers per CTCAE v5.0 criteria: Grade 1 (asymptomatic/mild symptoms requiring monitoring), Grade 2 (interruption of intervention ≤7 days with symptomatic treatment), Grade 3 (intervention termination and multidisciplinary consultation), and Grade 4 (immediate emergency care and permanent withdrawal).

All SAEs were reported within 12 h (life-threatening) to 24 h (non-critical). Full compliance with Council for International Organizations of Medical Sciences (CIOMS) VI standards for Suspected Unexpected Serious Adverse Reaction (SUSAR) reporting was maintained, with critical protocol amendments requiring ≥75% approval via anonymous voting.

### 2.11 Quality control and assurance

#### 2.11.1 Selection bias risks

To mitigate selection bias risks, this study implemented a rigorously designed RCT framework. Participants were stratified based on key baseline characteristics (e.g., age, disease severity) and subsequently allocated to intervention or control groups via computer-generated randomization sequences. This dual strategy of stratification and randomization ensured balanced distribution of confounding variables across study arms, thereby minimizing systematic differences between groups.

An independent statistician executed the randomization process to eliminate investigator interference in group assignment, while allocation concealment was maintained until participant enrollment to prevent selection bias arising from foreknowledge of treatment assignments. These measures collectively ensured inter-group comparability and mitigated potential biases associated with non-random allocation or investigator-driven participant selection.

#### 2.11.2 Control of confounding bias

Confounding bias predominantly arises during data analysis due to distorted causal relationships between exposure factors and outcome variables. When confounding factors (third variables associated with both exposure and outcome) remain inadequately adjusted, erroneous causal attribution may occur, leading to overestimation or underestimation of effect sizes. This study implemented multivariable regression models (adjusting for baseline covariates such as age and gender) combined with propensity score matching to statistically control known confounders.

Additionally, sensitivity analyses were conducted to assess potential impacts of unmeasured confounders, with *E*-values quantifying the robustness of causal inferences. A prospective covariate data collection protocol (based on literature review and causal diagram analysis) further ensured systematic identification of confounding factors.

#### 2.11.3 Design of the protocol

This study employed a prospective randomized double-blind RCT design, validated against international CONSORT guidelines. The randomization process is implemented through a computer-generated block randomization sequences. Participant allocation was executed by an independent statistician through an Interactive Web Response System (IWRS), ensuring allocation concealment until intervention initiation. A blind design (masking both investigators and participants) was implemented, with primary endpoints adjudicated by a blinded endpoint committee.

#### 2.11.4 Strict participant eligibility criteria

Implementing stringent inclusion criteria is a critical method for controlling bias. By establishing well-defined inclusion and exclusion criteria, the study population can be rigorously confined to a specific range, reducing heterogeneity among participants and facilitating objective conclusions regarding the observed factors.

#### 2.11.5 Blinding implementation and outcome assessment

Blinding procedures mitigate measurement bias and expectation bias by preventing awareness of intervention assignments among investigators and participants. This study implemented a three-tier blinding system: (1) Participant blinding (using sham stimulation and blinded the display screen); (2) Investigator blinding (group allocation concealed via central randomization system); (3) Statistician blinding (de-identified raw data processing). Primary endpoints were assessed by an independent Endpoint Adjudication Committee using standardized scales (e.g., NIHSS scores) under blinded conditions, with committee members excluded from trial operations and denied access to allocation databases.

#### 2.11.6 Laboratory quality assurance

All biomarker assays strictly adhered to ISO 15189:2022 medical laboratory standards, implemented through the “Clinical Biochemical Testing Standard Operating Procedures” (WS/T 641–2023). According to experimental quality control protocols, laboratory test results must be documented through computer-generated printouts to prevent data inaccuracies caused by human errors.

#### 2.11.7 Research training measures and methods

To ensure the smooth progress of the research, specialized clinical training sessions must be organized by the project team prior to the commencement of clinical trials. These sessions aim to provide uniform training for clinical researchers, including detailed instruction on the project implementation plan and operational procedures. Through these trainings, researchers are required to achieve comprehensive familiarity with the entire research process and specific implementation guidelines. This approach enhances intra-observer consistency and inter-observer consistency among researchers, thereby ensuring the reliability of clinical research conclusions.

#### 2.11.8 Participant compliance management measures

To enhance compliance and reduce participant attrition, the following strategies were implemented:

Ethical principle: Adherence to voluntary participation was ensured through signed informed consent forms.

Healthcare optimization: Patient retention was promoted by improving healthcare quality, treatment environment, and cost transparency to encourage continuous participation.

Physician-patient communication: Detailed explanations of diagnostic tests, therapeutic procedures, and follow-up requirements were provided to foster cooperation.

Contact documentation: Comprehensive contact information (including emergency contacts) was recorded for follow-up purposes.

Symptom monitoring: Participants received standardized symptom diaries with training on grading scales (e.g., Visual Analog Scale) to document disease progression and adverse events.

Treatment protocol compliance: Explicit instructions prohibited concomitant medications during the study. Completed symptom diaries were reviewed with participants at study closure to verify data accuracy.

#### 2.11.9 Data management quality control

Standardized personnel training, optimized questionnaire logic, and double-blind data entry protocols were implemented to enhance data completeness and accuracy, significantly reducing error rates and ensuring research outcome reliability.

#### 2.11.10 Data entry staff training

The study implemented a rigorous selection protocol for data verification and entry specialists, requiring candidates to hold at minimum a bachelor’s degree in medical sciences (including current master’s students) while demonstrating both professional responsibility and fundamental computer literacy. Selected personnel underwent systematic pre-service training encompassing five critical domains: questionnaire design rationale, database architecture, standardized coding protocols, data entry operational guidelines, and quality control specifications. This training program concluded with formal competency assessment and certification by an independent academic committee.

To ensure procedural standardization and data consistency, a dual-blind pilot verification and preliminary data entry phase was conducted prior to formal operations. This preparatory phase served to reinforce operational standardization through simulated practice while establishing unified quality evaluation benchmarks aligned with international research protocols.

## 3 Discussion

As a prevalent circadian rhythm sleep–wake disorder, CI has been shown to significantly impair quality of life, cognitive performance, and emotional regulation in affected individuals (28). While cognitive behavioral therapy for insomnia (CBT-I) remains the first-line intervention per international guidelines, its implementation faces substantial challenges including prohibitive healthcare costs, low adherence and shortage of certified therapists (29). Benzodiazepine receptor agonists (BZRAs), though effective in reducing sleep onset latency in short-term use, carry significant iatrogenic risks: withdrawal syndromes, dose-dependent daytime somnolence, cumulative cognitive impairment, and potential for misuse (30). Consequently, novel non-invasive neuromodulation techniques are emerging as promising alternatives, demonstrating superior safety profiles and precise targeting capabilities (31).

FD is a gastrointestinal dysfunction disorder characterized by chronic duration and persistent or recurrent upper abdominal symptoms, with rising incidence yet incompletely understood pathogenesis. Patients with overlapping somatic symptoms often exhibit aggravated clinical manifestations and diminished quality of life (32). Current therapeutic strategies designate proton pump inhibitors (PPIs) and H2 receptor antagonists as first-line treatments for epigastric pain syndrome (EPS), while prokinetic agents are preferred for postprandial distress syndrome (PDS). However, both approaches demonstrate suboptimal response rates and high recurrence (33). Notably, the adverse effects associated with long-term use of prokinetics, acid suppressants, and neuromodulators further compound the complexity of clinical management (34).

Chronic low-grade inflammation is implicated as a potential pathogenic pathway in CI. Patients with insomnia and shortened sleep duration exhibit elevated systemic C-reactive protein (CRP) levels, correlating with chronic inflammatory states (35). Reduction of proinflammatory cytokines has been shown to alleviate insomnia symptoms (36). Notably, low-grade inflammation also contributes to functional dyspepsia pathogenesis. Dyspeptic patients demonstrate low-grade duodenal mucosal inflammation (37), and attenuation of mucosal inflammation correlates with symptom improvement (38). Preclinical studies reveal that activation of the NF-κB signaling pathway and increased interleukin-6 (IL-6) levels in the duodenal mucosa characterize dyspepsia model rats, indicative of mucosal inflammation (39). Anti-inflammatory interventions in these models restore body weight, food intake, and small intestinal motility. Collectively, low-grade inflammation represents a shared pathogenic mechanism in CI and FD, positioning mucosal inflammation reduction as a potential therapeutic target for their comorbidity.

Enhanced vagal nerve activity serves as a critical therapeutic pathway for mitigating low-grade inflammation. Vagus nerve stimulation significantly reduces proinflammatory cytokine expression (40), and further mediates the downregulation of interleukin-6 (IL-6) in the duodenal mucosa of dyspeptic patients, correlating with decreased symptom severity indices (41). As a key immunoregulatory component, the vagus nerve modulates macrophage polarization to exert anti-inflammatory effects, which are abolished following surgical vagotomy (42). Experimental evidence demonstrates that vagal stimulation suppresses proinflammatory cytokine production in immature macrophages (39). In functional dyspepsia (FD) model rats, auricular vagus nerve stimulation downregulates serum interleukin-1β (IL-1β) levels, reduces visceral hypersensitivity, and alleviates dyspeptic symptoms (43). These findings collectively indicate that vagal neuromodulation attenuates low-grade inflammation through targeted cytokine suppression.

Clinical studies demonstrate that taVNS at varying intensities significantly reduces insomnia severity. A randomized controlled trial by Zhang et al. (44) revealed that taVNS (intensity: 0.8–1.5 mA, frequency: 4/20 Hz, pulse width: 200 μs) decreased Pittsburgh Sleep Quality Index (PSQI) scores in insomnia patients, accompanied by improved sleep quality, shortened sleep latency, and prolonged total sleep duration. Further validation by Wu X et al. (45) indicated that continuous taVNS intervention (intensity: 7–12 mA, frequency: 20 Hz, pulse width: 200 μs) significantly reduced neuronal activity in sensorimotor networks (right cerebellum, right medial superior frontal gyrus, and bilateral supplementary motor areas), with PSQI improvements surpassing those of the control group. In a randomized, double-blind, controlled trial, Dos Reis et al. (46) administered 20-min left-ear taVNS (20 Hz) and observed its positive effects on stress reduction and sleep quality enhancement. Research highlights the concha cymba as a potential optimal stimulation site for taVNS (47), yet no clear consensus exists regarding standardized reporting criteria. Current parameter combinations (frequency: 80 Hz, pulse width: 100 μs, duration: 40–60 s) require further clinical validation, though their multi-target regulatory effects on sleep–wake circuitry are mechanistically supported.

A randomized controlled trial demonstrated that 4-week twice-daily taVNS at 10 Hz or 25 Hz significantly improved symptom response rates and adequate relief rates in adults with Rome IV-defined functional dyspepsia compared to sham stimulation, with sustained efficacy through 12-week follow-up and no significant difference between frequencies, while exhibiting low adverse event incidence (48). The taVNS alleviates gastrointestinal dysfunction in patients with postprandial distress syndrome (49). Evidence indicates that auricular-vagal stimulation improves symptoms including early satiety, postprandial bloating, epigastric fullness, abdominal pain, heartburn, and nausea-vomiting in dyspeptic populations (50, 51), while enhancing quality of life, reducing anxiety-depressive comorbidity, and maintaining sustained therapeutic effects without adverse events. Mechanistically, taVNS enhances vagal efferent signaling to ameliorate dyspeptic symptoms (52), significantly lowering symptom severity scores and elevating quality-of-life indices, with durable long-term efficacy.

The taVNS demonstrates efficacy in ameliorating CI and comorbid dyspeptic symptoms without adverse effects, representing a promising therapeutic strategy for geriatric patients with overlapping CIa and FD, mechanistically linked to vagal-mediated modulation. Both CI and FD share immunoinflammatory pathogenic pathways (53), with clinical symptom alleviation observed following reduced inflammatory cytokine expression (54–56). Insomnia has been identified as a potential marker of subclinical inflammatory activity (57). While inflammation reduction is critical for treating CI comorbid with FD, the underlying mechanisms remain incompletely elucidated.

Accumulating evidence indicates that low-grade inflammatory status may serve as the fundamental pathological mechanism underlying the comorbidity of chronic insomnia and functional dyspepsia. Although current clinical research on taVNS has primarily concentrated on individual disorders, our preliminary clinical investigations have demonstrated its synergistic therapeutic efficacy in patients with comorbid chronic insomnia and functional dyspepsia, thereby providing direct clinical substantiation for this pathophysiological hypothesis. By implementing longitudinal monitoring of dynamic fluctuations in peripheral inflammatory markers and central neurotransmitters throughout taVNS intervention, this study systematically deciphers the bidirectional regulatory pathways of the auricular vagus nerve in peripheral immune modulation and central neurochemical equilibrium, yielding novel mechanistic evidence to elucidate the multimodal integrative regulatory functions of the vagus nerve.

This randomized double-blind sham-controlled trial comprised an active group receiving taVNS at the concha cymba (frequency: 80 Hz, pulse width: 100 μs, pulse 40–60s) for 30 min per session, 5 sessions weekly for 3 consecutive weeks, alongside the sham group undergoes identical procedures with non-active electrodes. Primary endpoints included variations in the PSQI and NDSI, with secondary outcomes encompassing ISI, SF-36, serum biomarkers (pro-inflammatory cytokines IL-1*β*, IL-4, IL-6, TNF-*α*, hs-CRP, TGF-β) and neurotransmitters (DA, 5-HT, NE, Glu, GABA). Should the trial demonstrate statistical superiority of taVNS over sham in both symptom domains, it will not only elucidate the neuroimmune axis mechanism underlying comorbid CI with FD in elderly patients (e.g., vagus nerve-mediated duodenal mucosal inflammatory regulation), but also propose a novel non-pharmacological intervention characterized by precision targeting, elevated safety, and optimal adherence, thereby establishing a neuromodulation-based integrative therapeutic strategy for geriatric comorbidity management.

This study acknowledges limitations inherent to its exploratory design. The modest sample size reflects both the prospective nature of the trial and stringent inclusion criteria for CI comorbid with FD. Although preliminary clinical evidence suggests the therapeutic efficacy of high-frequency taVNS, the absence of prior literature supporting this intervention introduces potential bias and elevates uncertainty in evidence quality. Current taVNS protocols lack expert consensus regarding optimal stimulation parameters (frequency, intensity, site), and resource constraints precluded comparative effectiveness analyses across parameter combinations. Furthermore, the absence of objective neuroimaging or electroencephalographic biomarkers in outcome measures may introduce measurement bias. These limitations will be systematically addressed in subsequent validation studies.
